# Dietary intakes of iron, folate, and vitamin B12 during pregnancy and correlation with maternal hemoglobin and fetal growth: findings from the ROLO longitudinal birth cohort study

**DOI:** 10.1007/s00404-023-06916-x

**Published:** 2023-01-28

**Authors:** David J. Rooney, Marie Conway, Linda M. O’Keeffe, Ciara M. McDonnell, Helena C. Bartels, Cara Yelverton, Ricardo Segurado, John Mehegan, Fionnuala M. McAuliffe

**Affiliations:** 1 UCD Perinatal Research Centre, School of Medicine, University College Dublin, National Maternity Hospital, Dublin, Ireland; 2 School of Public Health, University College Cork, Cork, Ireland; 3 Paediatric Endocrinology and Diabetes, Children’s Health Ireland, Children’s University Hospital, Dublin, Ireland; 4 UCD School of Public Health, Physiotherapy and Sports Science, University College Dublin, Dublin, Ireland

**Keywords:** Birthweight, Dietary iron, Fetal growth, Growth trajectories, Iron deficiency anemia, Hemoglobin

## Abstract

**Purpose:**

Dietary micronutrient intakes of iron, folate and vitamin B12 are known to influence hemoglobin. Low maternal hemoglobin (maternal anemia) has been linked to low birthweight and other adverse health outcomes in the fetus and infant. Our primary aim was to explore relationships between maternal dietary micronutrient intakes, maternal full blood count (FBC) parameters and fetal abdominal circumference (AC) and estimated fetal weight (EFW) growth trajectories. Secondarily, we aimed to assess relationships between maternal dietary micronutrient intakes, maternal hemoglobin values and placental weight and birthweight.

**Methods:**

Mother–child pairs (*n* = 759) recruited for the ROLO study were included in this analysis. Maternal dietary micronutrient intakes were calculated from food diaries completed during each trimester of pregnancy. FBC samples were collected at 13- and 28-weeks’ gestation. Fetal ultrasound measurements were recorded at 20- and 34-weeks’ gestation. Growth trajectories for AC and EFW were estimated using latent class trajectory mixture models.

**Results:**

Dietary intakes of iron and folate were deficient for all trimesters. Mean maternal hemoglobin levels were replete at 13- and 28-weeks’ gestation. Dietary iron, folate and vitamin B12 intakes showed no associations with fetal growth trajectories, placental weight or birthweight. Lower maternal hemoglobin concentrations at 28 weeks’ gestation were associated with faster rates of fetal growth and larger placental weights and birthweights.

**Conclusion:**

The negative association between maternal hemoglobin at 28 weeks’ gestation and accelerated fetal and placental growth may be due to greater consumption of maternal iron and hemoglobin by fetuses’ on faster growth trajectories in addition to placental biochemical responses to lower oxygen states.

## What does this study add to the clinical work

**Table:** 

While this study found no associations between dietary micronutrient intakes and fetal growth, we found that lower maternal hemoglobin concentrations at 28 weeks’ gestation were associated with faster rates of fetal growth and larger placental weights and birthweights. Based on these findings we postulate the ‘consumption theory’ of pregnancy: that fetuses on faster growth trajectories have a higher requirement for oxygen that results in a greater consumption of maternal iron and hemoglobin.	

## Introduction

Anemia is defined as the reduced oxygen carrying capacity of blood due to low serum hemoglobin concentrations [1]. Hemoglobin concentrations are influenced by dietary micronutrient intakes of iron, vitamin B12 and folate [1]. Iron deficiency is the most widespread nutritional disorder in the world affecting up to 50% of pregnant women [2, 3]. It is by far the most common cause of anemia and has been linked to adverse health outcomes in the mother, fetus and infant [4]. Maternal iron deficiency is a risk factor for low birthweight, pre-term birth, stillbirth and perinatal death [5]. Maternal iron deficiency also increases the risk of infant anemia during the first year of life and has been associated with impaired infant cognitive function [6, 7].

Previous research has found that folate deficiency is a cause of megoblastic anemia [8], and vitamin B12 deficiency leads to pernicious anemia [9]. Deficiencies of vitamin B12 and folate may be associated with lower birthweight infants [9, 10].

Understanding the interrelation between maternal dietary micronutrient intakes, maternal hemoglobin and fetal growth may provide insight into prevention of adverse health outcomes for the fetus and infant.

Universal iron supplementation in pregnancy has been adopted by the World Health Organisation and the International Federation of Gynaecology and Obstetrics [11, 12]. Current guidance in some countries including the United Kingdom (UK) and Ireland favour dietary prevention of iron deficiency and do not commit to universal iron supplementation for all women [4, 5, 13]. This may be due, in part, to evidence suggesting that iron supplementation for pregnant women at low risk of iron deficiency, some of whom will develop iron excess with supplementation, results in a higher incidence of hypertensive disorders of pregnancy, small for gestational age infants and smaller placental weights [14, 15].

UK and Irish guidance recommend the daily consumption of 14.8 mg dietary iron and 1.5 mg dietary vitamin B12 for women of childbearing age [16–18]. Dietary reference intakes (DRI) of folate for this cohort vary internationally from 200 to 400 mcg daily [16, 17, 19]. Globally, it is recommended that all women take an additional daily 400 mcg folic acid supplement in the preconceptual period and for at least the first 3 months of pregnancy [17, 19]. Further research is needed into associations of maternal intakes of iron, vitamin B12 and folate during pregnancy, hemoglobin concentrations during pregnancy and measures of fetal and neonatal growth, including fetal growth trajectories as growth trajectories allow for determination of longitudinal growth patterns.

Our primary aim was to assess differences between maternal dietary micronutrient intakes (iron, folate and vitamin B12), and maternal full blood count (FBC) parameters, according to fetal abdominal circumference (AC) and estimated fetal weight (EFW) growth trajectory classes. Secondarily, we aimed to assess associations between maternal dietary micronutrient intakes (iron, folate and vitamin B12), and maternal hemoglobin, with placental weight and birthweight.

## Material and methods

### Study participants

This study is a secondary analysis of 759 mother–child pairs from the Randomised cOntrol trial of LOw glycaemic index diet in pregnancy to prevent macrosomia (ROLO study), conducted at the National Maternity Hospital, Dublin between 2007 and 2011 [20]. Secundagravida women aged > 18 years old, with a singleton pregnancy ≤ 18 week’s gestation, who previously delivered an infant > 4 kg in their first pregnancy, were eligible to take part [20]. Women with known medical disorders and those taking any regular medications were also excluded, in addition to women with a current or past history of gestational diabetes [20]. Women were randomised to either the intervention group where they received low glycemic index dietary advice, or to the control group where they received routine antenatal care. More detailed methods and results from the ROLO study have been published previously [20, 21].

### Maternal measurements

Maternal height (m), weight (kg) and body mass index (kg/m^2^) were recorded at the first antenatal consultation (booking visit) at 13 weeks’ gestation (Table 1). Blood samples were also obtained at 13 weeks’ gestation and again at 28 weeks’ gestation. All bloods were processed to obtain serum samples, and serum subsequently analysed for full blood count (FBC) parameters, including hemoglobin, using the Sysmex XE-2100 fluorescence flow cytometry analyser. The results were displayed using the CliniSys WinPath laboratory information management system and accessed retrospectively for this secondary analysis. FBC reference ranges were derived from Dacie and Lewis Practical Haematology Edition 10 (2006) in line with local haematology policy [22]. Smoking status and supplement intake were self-reported by women. While nutritional supplement intake was recorded, the supplement type was not.

**Table 1:** Characteristics of the mother, fetus, neonate and placenta from the randomised control trial of low (ROLO) glycemic index diet in pregnancy

	*n* (%)	Mean	SD
Maternal characteristics			
Maternal age at delivery (years)	755	32.45	4.19
Gestational age at booking (weeks)^a^	758	12.95	2.31
Maternal height (cm)	756	165.99	6.42
Maternal weight at booking (kg)^b^	756	70.55	16.6
Maternal BMI at booking (kg/m^2^)^b^	755	25.67	5.43
Maternal BMI Category (booking)			
Underweight (< 18.5 kg/m^2^)	4 (0.5)	–	–
Normal (18·5–24·9 kg/m^2^)	320 (42.4)	–	–
Overweight (25–29.9 kg/m^2^)	288 (38.1)	–	–
Obese (> 30 kg/m^2^)	143 (18.9)	–	–
Ethnicity (*n* = 759)			
Caucasian	743 (97.9)	–	–
Other ethnicity	16 (2.1)	–	–
Education level (*n* = 627)			
Third level^c^	490 (78.1)	–	–
Less than third level	137 (21.9)	–	–
Smoking in pregnancy (*n* = 622)			
Yes	46 (7.4)	–	–
No	576 (92.6)	–	–
Supplement use in pregnancy (*n* = 626)			
Yes	356 (56.9)	–	–
No	270 (43.1)	–	–
Folic acid use in pregnancy (*n* = 625)			
Every/most days	411 (65.8)	–	–
Sometimes	178 (28.5)	–	–
Never	36 (5.8)	–	–
Delivery type (*n* = 700)			
Spontaneous vaginal	526 (75.1)	–	–
Other	174 (24.9)	–	–
RCT group (*n* = 759)			
Intervention	369 (48.6)	–	–
Control	390 (51.4)	–	–
Fetal characteristics			
AC fetal growth trajectory class (*n* = 750)			
Slow	215 (28.7)	–	–
Fast	535 (71.3)	–	–
EFW fetal growth trajectory class (*n* = 759)			
Very slow	30 (4)	–	–
Moderately slow	481 (63.4)	–	–
Moderately fast	226 (29.8)	–	–
Very fast	22 (2.9)	–	–
Neonatal and placental characteristics			
Gestational age at birth (days)^b^	754	282.50	11
Birthweight (g)	756	4029.43	475.28
Birthweight centile^b^	706	79.25	35.7
Birth length (cm)	634	53.00	3.0
Head circumference at birth (cm)^b^	634	35.70	1.5
Abdominal circumference at birth (cm)^b^	265	33.20	3.0
Placental weight (g)	644	770.64	169.12

*RCT* randomised controlled trial, *AC* abdominal circumference, *EFW* estimated fetal weight, *BMI* body mass index

^a^Booking: first antenatal clinic visit

^b^Data displayed as Median (IQR)

^c^Third level education: education from a higher education institute (universities, institutes of technology and colleges of education)

### Dietary intake

Dietary assessments were carried out for each trimester of pregnancy using comprehensive 3-day food diaries: one in the first trimester before the low glycemic index dietary intervention and one each in the second and third trimesters of pregnancy [20]. Dietary intakes were analysed using NetWISP nutritional analysis software version 3.0 (Tinuviel software, Llanfechell, Anglesey, UK). This software package utilised the food composition database from the sixth edition of McCance and Widdowson’s food composition tables and provided an indication of the amounts and types of foods eaten, as well as the nutritional content of the women’s diets [23].

### Measures of fetal growth

Fetal ultrasound measurements were obtained at 20- and 34-weeks’ gestation by one of two blinded ultrasonographers using a Voluson 730 Expert (GE Medical Systems, Germany) [20, 24]. AC and EFW were recorded. EFW was calculated using the Hadlock 4-parameter formula [25]. Infant measurements obtained at delivery included: birthweight, birth length, head circumference and abdominal circumference (Table 1). Placental weight was also recorded. Birthweight centiles were calculated using Gestation Network’s Bulk Calculator version 6.2.3 UK as described by Walsh et al. [20]. AC and EFW growth trajectory models were developed subsequently using latent class modelling as described by Bartels et al. [24]. Briefly, measures of AC and EFW collected at 20- and 34-weeks’ gestation, and weight and AC at birth were used to derive AC and EFW trajectories [24]. Two fetal growth trajectories were identified for AC: a “slow” trajectory and a “fast” trajectory [24]. For EFW, four trajectories were identified: a “very-slow” trajectory, a “moderately-slow” trajectory, a “moderately-fast” trajectory and a “very-fast” trajectory [24].

### Statistical analyses

All statistical analyses were carried out using IBM Statistical Package for the Social Sciences software version 26 for Mac. Individual descriptive data were presented as mean and standard deviation or median and interquartile range depending on normality. Independent-Samples *T* Tests and Analysis of Variance were used with bootstrapping to explore differences between maternal FBC and dietary micronutrient parameters and the AC and EFW growth trajectories. Linear regression was completed to analyse relationships between maternal dietary micronutrient intakes and FBC results with placental weight and birthweight. Several variables were controlled for in all regression models, including randomised control trial group, maternal body mass index at booking, maternal age at delivery, use of supplements during pregnancy and maternal education status (as a surrogate for socioeconomic status). Statistical significance was set at *P* < 0.05.

### Ethical approval

This study was Granted ethical approval and maternal written consent was obtained for all participants. Ethics Committee approval was granted by the National Maternity Hospital, Dublin in June 2006. The clinical trial registry number for the parent study is ISRCTN54392969 (http://www.isrctn.com/).

## Results

Baseline characteristics for the 759 mother–child pairs analysed in the ROLO study control and intervention groups are shown in Table 1. 65.8% of women reported taking folic acid almost daily during pregnancy. 56.9% of women reported using additional supplements during pregnancy, the specifics of which were not captured as part of the ROLO study. Overall dietary intakes of iron, folate and vitamin B12 were stable across all three trimesters of pregnancy (Table 2). While dietary intakes of vitamin B12 were replete in each trimester, dietary intakes of iron and folate showed similar levels of deficiency across all three trimesters (Table 2). Maternal hemoglobin showed a downward trend from early to late pregnancy (Table 2) with 1.7% of mothers having anemia in the first trimester, and 10.9% of mothers having anemia in the third trimester. Other FBC parameters did not show the same downward trend between early and late pregnancy.

**Table 2:** Maternal dietary intakes and full blood count results during pregnancy in ROLO mothers

	Trimester 1			Trimester 2			Trimester 3		
	*N* (%)	Mean	SD	*N* (%)	Mean	SD	*N* (%)	Mean	SD
Dietary intakes									
Dietary iron (mg)^a^	548	10.71	4.27	554	10.66	4.18	549	10.89	4.36
Deficient dietary iron intake (< 14.8 mg/day)	471 (85.9)	–	–	475 (85.7)	–	–	469 (85.4)	–	–
Sufficient dietary iron intake (≥ 14.8 mg/day)	77 (14.1)	–	–	79 (14.3)	–	–	80 (14.6)	–	–
Dietary Vitamin B12 (µg)^a^	548	3.84	2.49	554	4.10	2.58	549	4.21	2.53
Deficient dietary vitamin B12 (< 1.5 mcg/day)	29 (5.3)	–	–	19 (3.4)	–	–	16 (2.9)	–	–
Sufficient dietary vitamin B12 (≥ 1.5 mcg/day)	519 (94.7)	–	–	535 (70.5)	–	–	533 (97.1)	–	–
Dietary folate (µg)^a^	548	253.1	125.9	554	264.5	113.8	549	258.5	120
Deficient dietary folate (< 400 mcg/day)	499 (91.1)	–	–	508 (91.7)	–	–	492 (89.6)	–	–
Sufficient dietary folate (≥ 400 mcg/day)	49 (8.9)	–	–	46 (8.3)	–	–	57 (10.4)	–	–

	Early pregnancy (13 weeks)			Late pregnancy (28 weeks)		
	*N* (%)	Mean	SD	*N* (%)	Mean	SD
Full blood count results						
HgB (g/dL)	756	12.9	0.9	713	11.6	0.9
Anemia in early pregnancy (HgB < 11 g/dL) and late pregnancy (HgB < 10.5 g/dL)	13 (1.7)	–	–	78 (10.9)	–	–
Without anemia in early pregnancy (HgB ≥ 11 g/dL) and late pregnancy (HgB ≥ 10.5 g/dL)	743 (98.3)	–	–	635 (89.1)	–	–
HCT (%)	756	0.37	0.02	713	0.34	0.02
MCV (fL)^a^	756	86	4.6	713	88.1	5.3
MCH (pg)^a^	756	30	1.8	713	30.1	2.3
MCHC (g/dL)	756	34.73	0.95	713	34.07	0.95

*HgB* haemoglobin, *HCT* haematocrit, *MCV* mean cell volume, *MCH* mean corpuscular haemoglobin, *MCHC* mean corpuscular hemoglobin concentration

^a^Data displayed as Median (IQR)

No evidence of differences in dietary intakes of iron, vitamin B12 and folate between those infants with an AC on a “slow trajectory” compared to those on a “fast trajectory” were found. Mean differences in dietary iron intake between the AC “slow” and “fast” growth trajectories were negligible across all trimesters (Table 3). All FBC parameters were similar across the three trimesters for the slow and fast AC growth trajectories (Table 3). Similarly, there were no significant differences in dietary intakes of iron, vitamin B12 and folate between the four EFW growth trajectories of “very slow”, “moderately slow”, “moderately fast” and “very fast”. There was a 1 mg mean difference in dietary iron for all trimesters between the EFW growth trajectory classes but this did not reach statistical significance (Table 4). Mean FBC parameters were also similar across the EFW growth trajectory classes. We found small but statistically significant differences, between the moderately slow and moderately fast EFW trajectory classes and hemoglobin (*P* = 0.046) and haematocrit (*P* = 0.026) in late pregnancy (28 weeks) (Table 4).

**Table 3:** Differences in dietary intakes and full blood count results during pregnancy across the abdominal circumference growth trajectory class

	AC slow trajectory			AC fast trajectory			Mean (95% CI) difference*	*P*
	*n*	Mean	SD	*n*	Mean	SD		
Dietary intakes								
Trimester 1 dietary intakes								
Iron (mg)^a^	172	10.67	4.07	375	10.71	4.35	0.12 (− 0.53 to 0.82)	0.703
Vitamin B12 (µg)^a^	172	3.91	2.45	375	3.82	2.55	0.18 (− 0.19 to 0.57)	0.348
Folate (µg)^a^	172	247.19	116.86	375	257.42	131.08	− 14.10 (− 31.64 to 3.49)	0.124
Trimester 2 dietary intakes								
Iron (mg)^a^	173	10.58	3.81	380	10.70	4.40	− 0.03 (− 0.63 to 0.64)	0.931
Vitamin B12 (ug)^a^	173	4.00	2.68	380	4.15	2.56	− 0.04 (− 0.41 to 0.32)	0.832
Folate (ug)^a^	173	259.84	109.32	380	265.97	114.58	− 5.78 (− 22.21 to 11.23)	0.503
Trimester 3 dietary intakes								
Iron (mg)^a^	174	11.12	4.08	374	10.84	4.57	0.07 (− 0.53 to 0.69)	0.837
Vitamin B12 (µg)^a^	174	4.26	2.68	374	4.18	2.46	0.20 (− 0.31 to 0.79)	0.455
Folate (µg)^a^	174	254.51	99.95	374	261.10	125.66	− 8.23 (− 25.26 to 10.27)	0.387
Full blood count results								
Early pregnancy (13 weeks)								
HgB (g/dL)	215	12.81	0.83	532	12.88	0.89	− 0.07 (− 0.20 to 0.07)	0.313
HCT (%)	215	0.37	0.02	532	0.37	0.23	− 0.00 (− 0.01 to 0.00)	0.184
MCV (fL)	215	86.02	4.01	532	85.85	3.87	0.17 (− 0.45 to 0.81)	0.573
MCH (pg)^a^	215	30.00	1.6	532	30.00	1.8	0.10 (− 0.17 to 0.37)	0.468
MCHC (g/dL)	215	34.78	0.91	532	34.73	0.96	0.06 (− 0.10 to 0.20)	0.458
Late pregnancy (28 weeks)								
HgB (g/dL)	206	11.63	0.88	501	11.55	0.93	0.08 (− 0.08 to 0.22)	0.306
HCT (%)	206	0.34	0.02	501	0.34	0.02	0.00 (− 0.00 to 0.01)	0.442
MCV (fL)	206	88.15	4.52	501	87.70	4.12	0.45 (− 0.31 to 1.16)	0.237
MCH (pg)^a^	206	30.40	2.3	501	30.00	2.1	0.22 (− 0.08 to 0.52)	0.155
MCHC (g/dL)	206	34.13	0.94	501	34.05	0.95	0.08 (− 0.07 to 0.24)	0.315

Mean (95% CI) and *P* value for significant differences between groups determined using Independent-Samples *T* test with Bootstrap

*AC* abdominal circumference, *HgB* haemoglobin, *HCT* haematocrit, *MCV* mean cell volume, *MCH* mean corpuscular haemoglobin, *MCHC* mean corpuscular hemoglobin concentration

^a^Data displayed as median (IQR)

*Mean difference between slow and fast trajectories for abdominal circumference

**Table 4:** Differences in dietary intakes and full blood count results during pregnancy across the estimated fetal weight growth trajectory class

	EFW very slow				EFW moderately slow (reference group)			EFW moderately fast				EFW very fast				*P*
	*n*	Mean	SD	Mean (95% CI) difference^b^	*n*	Mean	SD	*n*	Mean	SD	Mean (95% CI) difference^b^	*n*	Mean	SD	Mean (95% CI) difference^b^	
Dietary intakes																
Trimester 1 dietary intakes																
Iron (mg)^a^	21	9.94	3.85	0.47 (− 1.97 to 2.37)	356	10.73	4.27	157	10.89	4.46	0.18 (− 0.45 to 0.80)	14	10.55	4.10	0.51 (− 1.22 to 2.11)	0.862
Vitamin B12 (µg)^a^	21	3.04	2.81	0.31 (− 0.88 to 1.31)	356	3.94	2.43	157	3.86	2.71	− 0.04 (− 0.44 to 0.37)	14	2.81	2.11	0.73 (− 0.04 to 1.45)	0.514
Folate (µg)^a^	21	212.19	166.21	25.12 (− 25.80 to 70.22)	356	249.94	126.39	157	260.24	123.90	1.16 (− 17.92 to 20.84)	14	249.60	138.06	7.41 (− 37.77 to 53.86)	0.751
Trimester 2 dietary intakes																
Iron (mg)^a^	21	9.95	4.33	1.15 (− 0.08 to 2.31)	360	10.54	4.09	159	10.97	4.76	− 0.01 (− 0.65 to 0.59)	14	10.78	3.92	− 0.09 (− 1.74 to 1.69)	0.523
Vitamin B12 (µg)^a^	21	3.78	1.90	0.59 (0.03 to 1.16)	360	4.10	2.61	159	4.14	2.73	− 0.06 (− 0.43 to 0.31)	14	4.11	2.58	− 0.02 (− 0.98 to 0.84)	0.593
Folate (µg)^a^	21	235.57	146.03	12.67 (− 30.37 to 53.45)	360	264.97	108.97	159	264.99	121.41	− 2.70 (− 20.72 to 15.35)	14	292.03	155.32	− 7.59 (− 52.61 to 38.66)	0.899
Trimester 3 dietary intakes																
Iron (mg)^a^	20	10.19	4.75	0.39 (− 1.05 to 1.71)	356	10.96	4.23	159	11.10	4.63	− 0.36 (− 1.06 to 0.30)	14	8.72	4.71	0.80 (− 2.08 to 3.23)	0.510
Vitamin b12 (µg)^a^	20	3.75	3.22	0.74 (− 0.06 to 1.51)	356	4.20	2.44	159	4.61	2.49	− 0.15 (− 0.63 to 0.35)	14	3.48	1.12	0.83 (− 0.31 to 1.92)	0.384
Folate (µg)^a^	20	230.89	84.41	15.05 (− 44.69 to 67.33)	356	258.32	117.48	159	262.44	120.60	4.15 (− 12.85 to 21.57)	14	290.46	177.67	− 5.46 (− 84.74 to 59.99)	0.904
Full blood count results																
Early pregnancy (13 weeks)																
HgB (g/dL)	30	12.92	0.94	− 0.06 (− 0.42 to 0.27)	480	12.86	0.89	224	12.84	0.81	0.02 (− 0.12 to 0.15)	22	12.65	1.32	0.21 (− 0.32 to 0.79)	0.710
HCT (%)	30	0.37	0.02	− 0.00 (− 0.01 to 0.01)	480	0.37	0.02	224	0.37	0.02	0.00 (− 0.00 to 0.00)	22	0.37	0.03	0.00 (− 0.01 to 0.02)	0.887
MCV (fL)	30	85.66	3.03	0.14 (− 0.93 to 1.24)	480	85.80	4.13	224	86.06	3.58	− 0.27 (− 0.85 to 0.32)	22	84.52	5.21	1.28 (− 0.68 to 3.60)	0.355
MCH (pg)^a^	30	29.90	1.2	0.11 (− 0.27 to 0.50)	480	30.00	1.9	224	30.00	2.0	− 0.11 (− 0.37 to 0.15)	22	29.80	2.0	0.75 (− 0.04 to 1.76)	0.133
MCHC (g/dL)	30	34.69	0.95	0.05 (− 0.27 to 0.42)	480	34.74	0.94	224	34.76	0.95	− 0.02 (− 0.18 to 0.14)	22	34.36	1.14	0.38 (− 0.08 to 0.90)	0.295
Late pregnancy (28 weeks)																
HgB (g/dL)	28	11.78	1.10	− 0.15 (− 0.54 to 0.26)	453	11.63	0.88	211	11.43	0.93	0.20 (0.06 to 0.35)	21	11.55	0.95	0.08 (− 0.34 to 0.49)	0.041*
HCT (%)	28	0.34	0.03	− 0.00 (− 0.01 to 0.01)	453	0.34	0.02	211	0.34	0.02	0.01 (0.00 to 0.01)	21	0.34	0.02	0.00 (− 0.01 to 0.01)	0.029**
MCV (fL)	28	88.18	3.46	− 0.34 (− 1.72 to 0.94)	453	87.84	4.41	211	87.75	4.16	0.09 (− 0.61 to 0.79)	21	86.67	3.89	1.17 (− 0.50 to 2.88)	0.628
MCH (pg)^a^	28	30.35	2.2	− 0.19 (− 0.72 to 0.35)	453	30.10	2.3	211	30.10	2.5	0.05 (− 0.25 to 0.36)	21	29.50	1.4	0.54 (− 0.00 to 1.22)	0.553
MCHC (g/dL)	28	34.19	1.13	− 0.11 (− 0.51 to 0.33)	453	34.08	0.91	211	34.05	1.00	0.03 (− 0.14 to 0.18)	21	33.93	0.81	0.15 (− 0.23 to 0.53)	0.784

Mean (95% CI) and *P* value for significant differences between groups determined using Analysis of Variance with Bootstrap

*EFW* estimated fetal weight, *HgB* haemoglobin, *HCT* haematocrit, *MCV* mean cell volume, *MCH* mean corpuscular haemoglobin, *MCHC* mean corpuscular hemoglobin concentration

*Significant at *P* < 0.05. Tukey post hoc analysis showed a significance of 0.046 between moderately slow and moderately fast trajectories

**Significant at *P* < 0.05. Tukey post hoc analysis showed a significance of 0.026 between moderately slow and moderately fast trajectories

^a^Data displayed as median (IQR)

^b^Mean difference between moderately slow (reference group) and very slow, moderately fast, and very fast trajectories for estimated fetal weight

Regression models were completed to determine associations between dietary intakes, maternal hemoglobin and placental weight, taking account of confounding variables (Table 5). There were no associations between dietary intakes of iron, folate or vitamin B12 and placental weight across the three trimesters of pregnancy. There was a negative association between placental weight at birth and maternal hemoglobin at 28 weeks’ gestation (B = − 0.103 (CI − 35.05, − 2.86) *P* = 0.021). Associations between dietary intakes, maternal hemoglobin and birthweight were also investigated using regression models (Table 5). There were no associations between dietary intakes of iron, folate or vitamin B12 and birthweight across the three trimesters of pregnancy. Birthweight and hemoglobin at 28 weeks’ gestation were found to be negatively associated (B = − 0.107 (CI − 95.43, − 13.10) *P* = 0.010).

**Table 5:** Associations between dietary intakes and full blood count results during pregnancy with placental weight and birthweight

	*B*	*P*	95% CI	
			Lower	Upper
Linear regression models for placental weight				
Trimester 1 dietary intakes				
Trimester 1 dietary iron (mg) vs. placental weight (g)	0.052	0.294	− 2.03	6.69
Trimester 1 dietary folate (µg) vs. placental weight (g)	0.026	0.605	− 0.11	0.19
Trimester 1 dietary B12 (µg) vs. placental weight (g)	0.005	0.921	− 7.34	8.12
Trimester 2 dietary intakes				
Trimester 2 dietary iron (mg) vs. placental weight (g)	− 0.025	0.606	− 5.68	3.32
Trimester 2 dietary folate (µg) vs. placental weight (g)	− 0.003	0.953	− 0.17	0.16
Trimester 2 dietary B12 (µg) vs. placental weight (g)	0.044	0.366	− 4.06	10.99
Trimester 3 dietary intakes				
Trimester 3 dietary iron (mg) vs. placental weight (g)	0.004	0.943	− 4.13	4.45
Trimester 3 dietary folate (µg) vs. placental weight (g)	0.028	0.574	− 0.10	0.19
Trimester 3 dietary B12 (µg) vs. placental weight (g)	0.008	0.873	− 5.05	5.94
Full blood count results				
Early HgB (g/dL) vs. placental weight (g)	− 0.050	0.249	− 25.62	6.66
Late HgB (g/dL) vs. placental weight (g)	− 0.103	0.021	− 35.05	− 2.86
Linear regression models for birthweight				
Trimester 1 dietary intakes				
Trimester 1 dietary iron (mg) vs. birthweight (g)	0.024	0.598	− 8.38	14.53
Trimester 1 dietary folate (µg) vs. birthweight (g)	0.014	0.755	− 0.33	0.46
Trimester 1 dietary B12 (µg) vs. birthweight (g)	0.003	0.941	− 19.06	20.56
Trimester 2 Dietary Intakes				
Trimester 2 dietary iron (mg) vs. birthweight (g)	0.044	0.321	− 5.78	17.59
Trimester 2 dietary folate (µg) vs. birthweight (g)	− 0.005	0.908	− 0.46	0.41
Trimester 2 dietary B12 (µg) vs. birthweight (g)	0.076	0.086	− 2.47	36.99
Trimester 3 dietary intakes				
Trimester 3 dietary iron (mg) vs. birthweight (g)	0.041	0.369	− 6.22	16.70
Trimester 3 dietary folate (µg) vs. birthweight (g)	0.012	0.783	− 0.33	0.44
Trimester 3 dietary B12 (µg) vs. birthweight (g)	0.046	0.301	− 6.66	21.47
Full blood count results				
Early HgB (g/dL) vs. birthweight (g)	− 0.023	0.560	− 54.17	29.37
Late HgB (g/dL) vs. birthweight (g)	− 0.107	0.010	− 95.43	− 13.10

Model adjusted for randomised control trial group, maternal body mass index at booking, maternal age at delivery, dietary supplement use in pregnancy and socioeconomic status (maternal education level)

*Early HgB* hemoglobin at 13 weeks’ gestation, *Late HgB* hemoglobin at 28 weeks’ gestation

## Discussion

In the current study, median dietary iron intakes were below the 14.8 mg DRI for all trimesters of pregnancy, and median dietary folate intakes were below the 400 mcg DRI across all three trimesters. Conversely, vitamin B12 intakes were above the 1.5 mcg DRI across all trimesters of pregnancy. Despite inadequate dietary intakes of both dietary iron and folate, maternal hemoglobin levels were replete at both 13- and 28-week’s gestation (Table 2). This is likely due to the fact that 56.9% of women used supplements during pregnancy, many of which may have been iron supplements. Additionally, 65.8% of women took supplemental folic acid in pregnancy.

No differences were found between dietary intakes of iron, folate or vitamin B12 between the various classes of the AC and EFW growth trajectories. Dietary data was collected using 3 day food diaries, and while food diaries are regarded as the gold standard for collection of dietary information, they are prone to reporting errors due to the burden on participants [26]. Mean maternal hemoglobin levels were higher in mothers carrying fetuses on moderately slow EFW growth trajectories compared to moderately fast EFW growth trajectories at 28 weeks’ gestation (Table 4). It should be noted that the majority of fetuses (*n* = 664) fell within these two trajectory classes while the other trajectory classes were small and inferences about them had lower power. We postulate that fetuses on faster growth trajectories have a higher requirement for oxygen and hemoglobin which may explain why maternal hemoglobin levels decreased across the four EFW growth trajectory classes in late pregnancy. In support of this theory, we found that neonates with higher birthweights were negatively associated with maternal hemoglobin levels at 28 weeks’ gestation (Table 5). This gives greater credence to the theory of oxygen and hemoglobin consumption by fetuses on a faster growth trajectory resulting in larger birthweights.

Maternal anemia has traditionally been seen as a risk factor for low birthweight and fetal growth restriction [27]. Studies have found lower birthweights in pregnancies with both low and high hemoglobin concentrations, thus highlighting an inverted U-shaped relationship [14, 28, 29]. The reasons for lower birthweights at the extremes of maternal hemoglobin concentrations appear to be intrinsically linked to placental growth responses to both low and high hemoglobin concentrations [14, 30]. Our findings of an overall negative association for birthweight and maternal hemoglobin at 28 week’s gestation may be relatable to the physiology of plasma volume expansion in pregnancy. We know that there is a maternal plasma volume expansion in pregnancy that leads to a reduction in hemoglobin concentration. Jwa et al. hypothesised that a reduction in maternal hemoglobin levels from early pregnancy to mid- or late pregnancy is a proxy for plasma volume increase during pregnancy that may be protective against adverse birth outcomes including delivery of a low birthweight or small for gestational age infant [31]. Previous studies have shown that the absence of an adequate increase in plasma volume in pregnancy is associated with lower birthweight and placental weights [32–34]. Jwa et al. showed that women with the least reduction in hemoglobin from early to late pregnancy had a significantly increased risk of delivering a low birthweight or small for gestational age infant compared to women with the intermediate or greatest reduction [31]. This may explain the negative association between late maternal hemoglobin and birthweight from our study—there was an approximate 1 g/dL mean drop in maternal hemoglobin from 13 to 28 weeks’ gestation (Table 2) which may have resulted in a suboptimal plasma volume expansion in our women throughout their pregnancies. It must be noted that there were significant differences between our own study and that of Jwa et al. The latter study had a Japanese cohort of women and maternal hemoglobin was collected at three timepoints in pregnancy in comparison to two timepoints in our own study [31]. Participants in the current study had optimal hemoglobin levels; however, further work in cohorts with suboptimal hemoglobin status is warranted. Additionally, our cohort was unique in that all of our women had previously given birth to a macrosomic infant and were thus more prone to delivering infants with larger birthweights in subsequent pregnancies based on both genetic and environmental factors.

Research has shown an association between maternal iron deficiency, maternal anemia and larger placental weights [28, 35]. Hemoglobin concentrations < 9 g/dL have been associated with the largest placental weights [14]. The mechanisms behind this association are not fully understood but studies have postulated a compensatory placental hypertrophy in response to maternal anemia [29]. We know that there is an upregulation of placental iron transport proteins such as ferroportin in placentas of anaemic mothers [36]. We also know that there is increased expression of angiogenic proteins such as vascular endothelial growth factor in the placentas of anaemic mothers which may be responsible for the hypertrophic placental response to maternal anemia [37]. On the other hand, high maternal hemoglobin concentrations have been associated with low placental weight [14]. It has been suggested that high hemoglobin concentrations may cause blood hyperviscosity, vascular occlusion and capillary congestion that may result in reduced availability of oxygen in the intervillous space and thereby suboptimal placental growth [14, 30]. Mitsuda et al. found a U-shaped relationship between maternal hemoglobin concentration and placental weight [29]. Placental weights decreased with increasing serum hemoglobin concentrations up to 12 g/dL and then increased thereafter [29]. In our study, mean hemoglobin concentrations at 28 week’s gestation were just below 12 g/dL (Table 2) which may explain our finding of a negative relationship between late maternal hemoglobin and placental weight (Table 5).

This study had a number of strengths. To our knowledge it is the first study of its kind to specifically look at the association between maternal full blood count indices and fetal growth trajectories. Several important confounders such as randomised control trial group, maternal age, body mass index, supplement use and socioeconomic status were included in regression analyses. All of these confounders have known associations with fetal and neonatal measures of growth. This study had a number of limitations. All women analysed as part of this study had previously given birth to a macrosomic infant. This makes it more difficult to relate the findings to the general pregnant population. While nutritional supplement intake was recorded, the supplement name was not so it is not known if supplements containing iron were consumed by women participating in this study. The current study includes FBC data only—inclusion of other biomarkers of iron status, such as serum ferritin and serum transferrin receptor, would facilitate a more complete analysis of the interplay between iron intakes, hemoglobin and fetal growth. The use of linear regression models in our study failed to address the possibility of potential non-linear relationships for our cohort—other studies have shown a non-linear relationship between maternal hemoglobin concentration and placental weight and birthweight [29]. Lastly, the trajectories used in this analysis were exploratory and need careful replication or refinement in similar populations.

## Conclusion

Dietary intakes of iron, folate and vitamin B12 showed no association with fetal AC and EFW growth trajectory classes, birthweight or placental weight. Lower maternal hemoglobin concentrations at 28 weeks’ gestation were associated with faster rates of fetal growth and larger placental weights and birthweights. These findings may be due to biochemical responses in the placenta to lower oxygen states, physiological protective expansions in plasma volume later in pregnancy and potentially greater consumption of maternal iron and hemoglobin by fetuses on accelerated growth trajectories.

## Data Availability

The authors confirm that all relevant data supporting the findings of this study are included within the paper. Any additional data required may be requested from the corresponding author.

## Abbreviations

AC: Abdominal circumference
DRI: Dietary reference intakes
EFW: Estimated fetal weight
FBC: Full blood count
ROLO: Randomised cOntrol trial of LOw glycaemic index diet in pregnancy to prevent macrosomia
UK: United Kingdom
